# Improving the response to future pandemics requires an improved understanding of the role played by institutions, politics, organization, and governance

**DOI:** 10.1371/journal.pgph.0001501

**Published:** 2023-01-20

**Authors:** Peter Berman, Maxwell A. Cameron, Sarthak Gaurav, George Gotsadze, Md Zabir Hasan, Kristina Jenei, Shelly Keidar, Yoel Kornreich, Chris Lovato, David M. Patrick, Malabika Sarker, Paolo Sosa-Villagarcia, Veena Sriram, Candice Ruck

**Affiliations:** 1 School of Population and Public Health, University of British Columbia, Vancouver, Canada; 2 Shailesh J. Mehta School of Management, Indian Institute of Technology Bombay, Mumbai, India; 3 Curatio International Foundation, Tbilisi, Georgia; 4 Johns Hopkins Bloomberg School of Public Health, Baltimore, Maryland, United States of America; 5 Tel Aviv University, Tel Aviv, Israel; 6 British Columbia Center for Disease Control, Vancouver, Canada; 7 James P. Grant School of Public Health, BRAC University, Dhaka, Bangladesh; 8 Instituto de Estudios Peruanos, Lima, Peru; Emory University School of Medicine, UNITED STATES

The COVID-19 pandemic has resulted in a global crisis that continues to challenge the resilience of health systems worldwide. Although most jurisdictions had timely access to knowledge emerging globally, there was great diversity in actual responses–including timing, choice of action, and intensity of implementation—across different jurisdictions, with resulting variations in health and social outcomes. As countries emerge from the pandemic, global efforts are taking shape to use the lessons from COVID-19 to improve preparedness for future pandemics. Although many assessments have already been made regarding overall national performance in responding to COVID-19, these have mostly focused on more ‘downstream’ actions and factors such as measures taken to reduce infection, clinical approaches to disease management, and technical capacity and have overlooked the ‘upstream’ forces that shaped and drove those responses. However, for the proposed reform initiatives to be effective will require a more in-depth understanding of the ‘upstream’ factors that drove the wide variation in responses to COVID-19. To address this, an interdisciplinary team at the University of British Columbia has proposed a framework to unite scholarship into the institutional, political, organizational, and governance (IPOG) aspects of the COVID-19 response.

One of the most notable features of the response to COVID-19 has been how actual responses had little association with prior technical assessments of health systems’ preparedness and capacities, even in countries at similar economic levels. Others have observed the extent to which the outcomes of the real-world response differed from pre-pandemic rankings conferred by preparedness indicators such as the Global Health Security Index (GHSI) [1] and the Joint External Evaluations under the International Health Regulations [2], suggesting that factors influencing the effectiveness of real-world pandemic responses were not captured well by these approaches. While large differences in financial and physical resources were presumed to be determinative, analyses have shown that differences in policy interventions were more heavily responsible than socio-economic differences for the wide range in mortality figures between nations [3]. The available evidence suggests that the vastly different experiences of many countries were driven largely by the wide range of contextual factors and social determinants that shaped countries’ response strategies to COVID-19 [4]. Given this diversity in responses, implementation, and subsequent health outcomes, it is apparent that there is a need to focus more inquiry on those key ‘upstream’ factors, such as institutional norms, processes of governance, politics, and the organization of health systems including the elements tasked with public health response, that influence effective decision-making and response during health emergencies such as COVID-19. These upstream factors must be better understood and the knowledge thus gained applied in improving preparedness for future public health crises.

Even prior to the pandemic, awareness was growing of how vital governance is in the function of health systems. Shortcomings in performance linked to governance exacerbate inequality. A critical feature of effective governance is that it can enable better performance even in the absence of good leadership, and can function as a defense in the face of poor leadership. More recently, there has already been substantial acknowledgment of the role that governance has played in pandemic responses. COVID-19 clearly demonstrated that public health capacity alone is not a guarantor of a robust crisis response, and that governance has been a key determinant of an effective pandemic response [5].

The influence of political factors has also been identified as a driver of pandemic responses. Researchers have assessed how the pandemic response differed between centralized and decentralized political systems [6], as well as between more democratic and more authoritarian systems [7]. Political partisanship has also been identified as a driver of pandemic responses at the level of both governments and individuals.

What has been largely overlooked, however, is that politics and governance do not exist in a vacuum, but rather influence and are influenced by other factors such as institutional norms and the structure and functioning of key organizations tasked with public health response. There are major gaps in our research here as well, such as the absence of system-level and comparable descriptors for organizational structure. For example, it has been acknowledged that South Korea’s institutions, shaped and reformed in the aftermath of MERS, have been instrumental to that nation’s successful response to COVID-19 [8]. Recent calls to expand National Public Health Institutes in support of future pandemic responses need further study. A recent scoping review of NPHI’s cited the paucity of systematic research available on this topic and identified several specific areas that would benefit from more in-depth investigation [9]

**Fig 1:**
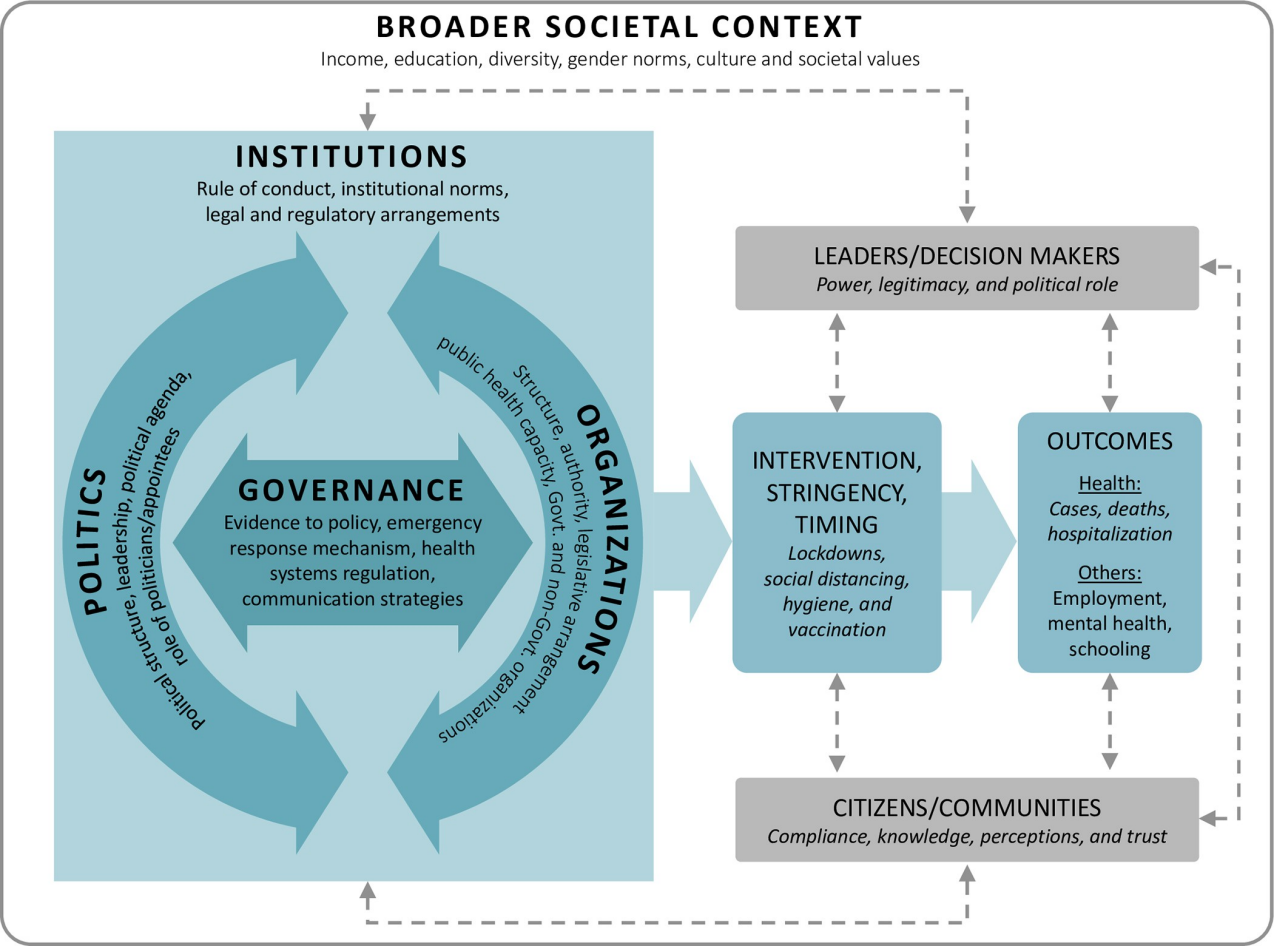
An analytical framework for investigating the impact of institutions, politics, organizations, and governance on the response to COVID-19. (Brubacher LJ, Hasan MZ, Sriram V, et al. Investigating the Influence of Institutions, Politics, Organizations, and Governance on the COVID-19 Response in British Columbia, Canada: a Jurisdictional Case Study Protocol).

What is particularly interesting is that, although institutions, politics, governance, and public health organization have all been given some degree of individual consideration in prior analyses of the pandemic response, research has seldom incorporated a broader view of the interconnectedness of these upstream factors and the need to consider that in future response design. It is our contention that COVID-19 has exposed the need to expand, deepen, and sharpen the focus of investigation to explore the intersection of all of these key contextual factors and how they combine to influence outcomes. This assertion drove the development of our IPOG analytical framework, the details of which have been published elsewhere [10]. We have applied this framework, elaborated on in Fig 1, with colleagues in multiple jurisdictions both within Canada as well as internationally. We posit that by incorporating these upstream factors into a single analytical framework, we can better understand how they interact with and influence one another in the development and deployment of a pandemic response. It would then be possible to propose improvements that could enable better responses to future pandemic threats and other health emergencies.

Although we initially elaborated and applied this framework to jurisdictions’ responses to COVID-19, we are encouraged by the relevance of the intersecting role of these factors to improving understanding of the social response to other health needs. Global responses, such as the weak capacities of multinational organizations to assure vaccine equity across countries during COVID-19, can be linked to the interface between global organization structures and national political dynamics. Emerging population health needs such as those related to mental health and substance use or food and diet-related causes of chronic disease epidemics require a broader view of relevant organizational actors and political channels of influence on policy and implementation. We feel these and other domains of emerging policy action would benefit from greater attention to the linkages we have explored that enhance more narrow discipline-based modes of enquiry.

COVID-19 has prompted governments around the world to reflect on how to better prepare for the next pandemic. Increasingly, it is evident that to be truly successful, future preparations must also be designed to address the contextual factors that influence societal responses. Greater attention is needed to how IPOG factors have influenced the range of responses and outcomes or how evidence on these factors can support proposed reforms. This requires incorporating these considerations in ongoing planning for new investments in preparedness and resilience. This must be supported by broadening the scope of research to include these factors. However, nearly two and a half years into the pandemic, what we see instead is a desire among societies to move on without fully understanding how we got here or what reforms are necessary to improve future health system resilience and prevent similar outcomes when the next pandemic strikes. We need to learn more about IPOG and how these factors can be managed to improve future preparedness and outcomes. We strongly urge that the current efforts to strengthen and invest in preparedness, such as those proposed recently by the G20 and the new Financial Intermediary Facility at the World Bank, along with the next iterations of the International Health Regulations, explicitly incorporate analyses of IPOG factors in national and sub-national settings. As governments seek to improve preparations for future pandemics, we ignore these considerations at our peril.
